# Progress in Surgery

**Published:** 1898-12-10

**Authors:** 


### PROGRESS IN SURGERY.

### PROGRESS IN GYNÆCOLOGY AND OBSTETRICS.

(*Continued from page* 165.)

**Extra-uterine Pregnancy.**— Tubo-uterine pregnancy Taylor considers perhaps the most dangerous of all. He thinks that in the older statistics it is highly probable that some tubo-ligamentary and cornual pregnancies have been mistaken for tubo-uterine, and points out that in a doubtful case the position of the round ligament is of service in deciding its character; the ligaments should be on the uterine side of a tubal or tuba-ligamentary pregnancy, while it should be on the other side or in the anterior wall of an interstitial or cornual pregnancy. In discussing the diagnosis of early rupture, Taylor says that the symptoms—those of internal hæmorrhage—are of most importance, next the history, and least the physical signs, as these are often uncertain and practically wanting. He summarises the elements which go to form a positive diagnosis of extra-uterine pregnancy, as follows: (1) A patient within the child-bearing limits of age, and one in whom a pregnancy is possible; (2) she has recently been in good health; (3) it is more likely than not that several years have passed since the last pregnancy; (4) there is a history of amenorrhœa accompanied or followed by (5) irregular uterine hæmorrhage, dark in colour, moderate in amount, and persistent in its course; (6) with this there may be the passage of some membrane, either as a complete decidua or in pieces; (7) on examination pulsating vessels may be felt in the vaginal vault on one side of the uterus; (8) on this side also and closely investing the back of the uterus there is nearly always a tubal tumour; (9) this tumour enlarges markedly and suddenly by recurrent hæmorrhages and by the formation of a hæmatocele directly continuous with the original tubal tumour; (10) these hæmorrhages are signalised by sudden spasms of severe abdominal pain, and by transient attacks of peritonitis; (11) the uterus is displayed by the hæmatocele at first backwards, and afterwards to the opposite side of the pelvis; (12) the uterus throughout is slightly enlarged, and may be proved to be empty.

Extra-uterine pregnancy may be mistaken for (1) pyosalpinx with amenorrhœa; (2) myoma; (3) simple abortion; (4) retroflexion of the gravid uterus; (5) antiflexion of the gravid uterus; (6) twisted pedicle tumours; (*a*) of the tube; (*b*) of the ovary.

Advanced abdominal and tubo-ligamentary pregnancy must be diagnosed from (1) normal intra-uterine pregnancy; (2) pregnancy in one half of a double uterus; (3) pregnancy in one horn of a bicornuate uterus. In tubo-abdominal pregnancy at full term it is sometimes difficult to distinguish this condition from an intra-uterine pregnancy when the walls of the uterus are unusually thin, but if the fundus of the uterus cannot be found apart from the pregnancy, this should excite strong suspicion against the extra-uterine position of the child.

The treatment of extra-uterine pregnancy is essentially operative. Werth considers that ectopic gestation must be considered as a malignant new growth, and therefore should be removed by operation at every stage of its development. Taylor points out that there are one or two exceptions to this sweeping assertion, *e.g.*, in the latter half of a tubo-abdominal or tubo-ligamentary pregnancy, when no sign of danger is present, it may be better practice to wait for operation until a time at or about the usual period of delivery when the child is viable and strong, than to operate earlier when the life of the child is certain to be sacrificed; again, occasionally here and there a case of ruptured tubal pregnancy or tubal mole with peri-uterine hæmatocele recovers without operation, but the natural cure is rarely very satisfactory, and contrasts unfavourably with operative methods.

In discussing the different methods of operating, Taylor says whenever there is diffuse bleeding, abdominal section is the best operation. In true retro-uterine hæmatocele, in which the pouch of Douglas is distended with blood, vaginal cœliotomy is indicated. Anterior vaginal cœliotomy has been extensively used during recent years in the treatment of peritubal hæmatocele due to tubal pregnancy; the main objections to this as a routine method of treatment are the following: (1) Occasional insufficient space for operative work: (2) frequent inability to remove thoroughly and cleanly all products of the misplaced pregnancy; (3) inability to wash out the abdomen satisfactorily; (4) inability to drain through the anterior opening; (5) occasional inability to extract the uterus without injury, that organ being enlarged and softened by the changes consequent on the associated pregnancy, so that Taylor thinks that abdominal section is still the better route for operative work in every case in which a posterior colpotomy is inapplicable or insufficient.

J. Berry Hart has recently described a method of operating in intact anterior tubo-ligamentary pregnancy, in which the fœtus is removed through an extra-peritoneal wound, the sac packed with iodoform gauze, and the placenta allowed to separate and come away slowly with the discharge.

In tubo-abdominal pregnancy at term there is no difficulty in the removal of the child by abdominal section; the crux of the operation is the treatment of the placenta, and this, Taylor maintains, should always be removed, as if left it will sooner or later almost invariably become septic.

**Uterine Displacements.**—Hammond[7] has written a paper on uterine displacements, in which he contends that uncomplicated flexions and versions give rise to no symptoms, and are not pathological. He says that the diseases attending the uterus in its new position are only those that occur in its more common position, usually being some perimetritic or parametritic process going on in the surrounding cellular and connective tissues of the vaginal vault and cervix uteri; that the symptoms produced by these diseases are the same whether the uterus is displaced or not, and that if these complications are absent, the displacement gives rise to no symptoms, and is often only discovered accidentally. Prolapse, as distinguished from retroflexion and anteflexion, he believes to be a true displacement giving rise to symptoms distinctly referable to and arising from its altered position. Fothergill[8] also writes supporting the view that flexions and versions of the uterus bear no causal relation to the symptoms, and points out that in each case treatment should be directed towards the removal of the cause more than towards the rectification of the flexion or version.

Nolle,[9] of Philadelphia, says that in most cases of retroflexion the displacement is really a secondary matter, the essential features being disease of the appendages, and for treatment he advocates extirpation of the appendages and hysterectomy. Smith[10] believes the best operation for the cure of displacement of the uterus is by vaginal shortening of the round ligaments, which has the following advantages: (1) It is very easy; (2) it can be done very quickly; (3) it should have no mortality; (4) it is free from subsequent pains; (5) it will do as well for cases of retroversion with fixation as for those in which the uterus is freely movable; (6) it does not interfere at all with subsequent pregnancy and labour; (7) it does not leave any painful wound or unsightly scar.

**The Treatment of Fibro-myomata of the Uterus.**—Parsons[11] says that of the various drugs used at the present time ergot is still the most valuable, being superior to hydrastis canadensis. He mentions five cases treated with thyroid extract; in four of these no appreciable diminution in size followed, but in the fifth there was a distinct reduction in size. Extract of mammary gland[12] has also been used with success, the hæmorrhage in many cases having ceased, the general health improved, and in some instances the tumour became smaller. When menorrhagia persists and increases in spite of medicines, Parsons recommends swabbing the uterine cavity with liq. ferri. perchlor. fort. after previous dilatation, and removal of the diseased endometrium with the curette. If these means fail he recommends electricity, quoting cases where marked improvement followed its use. He says that in suitable cases with electricity hæmorrhage can be stopped, the pressure symptoms and pain can be got rid of, and the tumour reduced in size, and this without the patient having to run any appreciable risk or suffer any mutilation of her sexual organs; he points out that the following cases are unsuitable for this treatment: (1) Fibro-cystic tumours growing rapidly in young subjects; (2) old hard tumours composed chiefly of fibrous tissue; (3) associated disease of the tubes and ovaries; (4) very large tumours. In all these conditions the question of abdominal section must be faced when there are severe and urgent symptoms. There are four procedures which can be adopted according to the case before us: Myomectomy, enucleation, castration, and hysterectomy. Myomectomy is only feasible with subperitoneal tumours which have become more or less extended from the uterine tissue. When the tumour is embedded in the uterine wall and we wish to save the sexual organs, enucleation must be the method adopted, but owing to the mortality in this operation being higher than hysterectomy it has been to a great extent discarded. Castration is not always feasible, nor are the results always satisfactory, the chief point in operating being not to leave a scrap of ovarian tissue behind and to remove the tubes as well. When it s not possible to remove the ovaries, hysterectomy must be done. He points out that hysterectomy still has a higher mortality than any other operation in gynæcology, while it is performed for a disease which in these cases if left alone is very rarely fatal, and which will in all probability cure itself in middle age.

Jessett[13] records three cases of fibro-myomata of the uterus complicating pregnancy in which he successfully performed hysterectomy, and says that, when comparing the mortality after pan-hysterectomy with those of induced abortion or premature labour, the statistics are so much in favour of the former method of treatment that the surgeon should not hesitate to recommend hysterectomy in all those cases in which the symptoms are such that surgical interference is imperatively indicated.

Bland Sutton[14] considers that uterine myomata might destroy life in many cases as surely as malignant tumours, though more slowly. He does not recommend enucleation, which has been found by continental operators to be attended with more disastrous results than hysterectomy. He thinks the four principal objections to it are: (1) The danger of oozing; (2) the length of the operation; (3) the protracted convalescence; (4) the resulting sinus. He believes that in cases requiring operation hysterectomy without removal of the ovaries is the best treatment, that the woman is better off with two ovaries and no uterus than with a uterus and no ovaries.

Howard Kelly[15] thinks that myomectomy is the operation of election in the treatment of uterine fibroids in women under 40 years of age, except in cases in which the tumours were interstitial and larger than a six months' pregnancy, or when there was extensive lateral inflammatory disease. Hystero-myomectomy was to be preferred to myomectomy after the fortieth year, except when the myomectomy was a simple operation as in pedicle tumours.

Gouilland[16] advocates ligation of the uterine arteries for the treatment of uterine fibroids, and quotes a number of cases in which the hæmorrhage ceased and the tumour diminished in size.

7 Med. Rec., June 4, 1898. 8 Practitioner, May, 189 8. 9 Med. Rec., July, 1898. 10 Med. Chron., Aug., 1898. 11 Lancet, July 23, 1898. 12 Med. Rec., June 4, 1898. 13 Lancet, Sept. 24, 1898. 14 Brit. Med. Jour., May, 1898. 15 Med. Rec., June 4, 1898. 16 Brit. Med Jour., July 2, 1898.

